# Promoting smoking cessation in Bangladeshi and Pakistani male adults: design of a pilot cluster randomised controlled trial of trained community smoking cessation workers

**DOI:** 10.1186/1745-6215-10-71

**Published:** 2009-08-14

**Authors:** Rachna A Begh, Paul Aveyard, Penney Upton, Raj S Bhopal, Martin White, Amanda Amos, Robin J Prescott, Raman Bedi, Pelham Barton, Monica Fletcher, Paramjit Gill, Qaim Zaidi, Aziz Sheikh

**Affiliations:** 1 UK Centre for Tobacco Control Studies, Primary Care Clinical Sciences, University of Birmingham, Birmingham, B15 2TT, UK; 2 Psychological Sciences, Institute of Health and Society, University of Worcester, Worcester, WR2 6AJ,UK; 3 Public Health Sciences Section, Centre for Population Health Sciences, The University of Edinburgh, Medical School, Teviot Place, Edinburgh, EH8 9AG, UK; 4 Centre for Translational Research in Public Health, Institute of Health & Society, Newcastle University, NE2 4HH, UK; 5 UK Centre for Tobacco Control Studies, Public Health Sciences Section, Centre for Population Health Sciences, The University of Edinburgh, Medical School, Teviot Place, Edinburgh, EH8 9AG, UK; 6 International Centre for Child Oral Health, King's College London, 26-29 Drury Lane, London, WC2B 5RL, UK; 7 Health Economics, University of Birmingham, Birmingham, B15 2TT, UK; 8 Education for Health, 10 Church Street, Warwick, CV34 4AB, UK; 9 British Heart Foundation, 4 Fitzhardinge Street, London, W1H 6DH, UK; 10 Allergy & Respiratory Research Group, Centre for Population Health Sciences, The University of Edinburgh, Levinson House, 20 West Richmond Street, Edinburgh, EH8 9DX, UK; 11 CAPHRI, University of Maastricht, the Netherlands

## Abstract

**Background:**

The prevalence of smoking is higher among Pakistani and Bangladeshi males than among the general population. Smokers who receive behavioural support and medication quadruple their chances of stopping smoking, but evidence suggests that these populations do not use National Health Service run stop smoking clinics as frequently as would be expected given their high prevalence of smoking. This study aims to tackle some of the main barriers to use of stop smoking services and adherence to treatment programmes by redesigning service delivery to be more acceptable to these adult male populations. The study compares the effectiveness of trained Pakistani and Bangladeshi smoking cessation workers operating in an outreach capacity ('clinic + outreach') with standard care ('clinic only') to improve access to and success of National Health Service smoking cessation services.

**Methods/design:**

This is a pilot cluster randomised controlled trial based in Birmingham, UK. Super output areas of Birmingham will be identified in which more than 10% of the population are of Pakistani and/or Bangladeshi origin. From these areas, 'natural geographical communities' will be identified. Sixteen aggregated agglomerations of super output areas will be identified, separating areas from each other using buffer regions in order to reduce potential contamination. These natural communities will be randomised to 'clinic + outreach' (intervention) or 'clinic only' (control) arms. The use of stop smoking services and the numbers of people quitting smoking (defined as prolonged self-reported abstinence at four weeks, three months and six months) will be assessed in each area. In addition, we will assess the impact of the intervention on adherence to smoking cessation treatments and patient satisfaction.

**Trial registration:**

Current Controlled Trials ISRCTN 82127540.

## Background

### The problem being addressed

The UK has a national network of National Health Service (NHS) smoking cessation services offering a range of interventions proven to be effective in facilitating smoking cessation. Those using these cessation services are four times more likely to succeed in quitting than those who attempt to stop smoking on their own[1]. Bangladeshi and Pakistani males, who have a high prevalence of smoking[2] are however half as likely as the White population to use these services[3,4]. This may be because Bangladeshi and Pakistani smokers are poorly served by current smoking cessation services. One possibility is that existing service formulations need to be adapted to better meet the needs of these minority populations.

This study aims to provide key components of the evidence base for promoting effective smoking cessation in these two large minority ethnic communities comprising over one million UK citizens (1.8% of the UK population)[5]. Bangladeshis and Pakistanis are two of the most socio-economically deprived and marginalised communities in the UK with persistent poor health outcomes for a range of long-term disorders. Stopping smoking is especially important in these populations as Pakistani and Bangladeshi groups are at significantly increased risk of heart disease, stroke and type 2 diabetes when compared to the White population[6,7]; stopping smoking would reduce the risk of these conditions by at least one-third[8,9].

We have been pursuing a phased programme of work[3,10,11], in line with the Medical Research Council's (MRC) complex intervention framework, studying smoking behaviour in these communities which we seek to advance to a definitive (Phase III) trial stage [12-14]. The work reported here seeks to refine and pilot a theoretically informed, culturally acceptable, trained community smoking cessation worker model of care suitable for subsequent formal evaluation in a multi-centre cluster randomised controlled trial[12].

We are focusing on these two ethnic groups because of the high prevalence of smoking, the shared religious tradition[15], the high disease burden from cardiovascular, cerebrovascular and respiratory disorders[16], and our collective expertise in researching these communities. It is anticipated that the insights gleaned from in-depth work with these populations will provide important transferable lessons for future smoking cessation work in other minority ethnic groups.

### Smoking prevalence in ethnic minority communities

Data from the 2004 Health Survey for England revealed marked ethnic and gender variations in smoking patterns, with cigarette smoking prevalence being particularly high amongst Bangladeshi men (40%, compared with the national average of 24%)[2]. Smoking prevalence in Pakistani males (29%) was somewhat higher than the national average. This was in stark contrast to smoking figures in Pakistani (5%) and Bangladeshi (2%) women, which were significantly lower than the national average for women (23%). Similarly, smoking prevalence in males was particularly high in Bangladesh and Pakistan[17].

The English national smoking cessation services collect data quarterly on ethnicity using very broad ethnic groupings. Although 2.3% of English smokers are 'Asian'[4], only 1% of clinic users were classified as being 'Asian'[18], raising uncertainty over whether the national network of services is able to attract and meet the needs of these populations. Parts of Birmingham (and some other cities in the UK) have begun to develop models of care similar to those that are proposed in this study; these are however being implemented on a somewhat ad hoc basis, without clear evidence of effectiveness, and without rigorous evaluation.

Systematic reviews and meta-analyses have found that behavioural (counselling)[19] and some pharmacological interventions [20-22] increase the likelihood of successful smoking cessation and these findings have been incorporated into evidence-based guidelines for smoking cessation [23-25].

Although increasing smoking cessation remains a national priority[26], these effective interventions are underused within ethnic minority communities[3]. There is thus at present little evidence on how effectively to facilitate smoking cessation in Bangladeshi and Pakistani communities. There has, perhaps as a consequence, been few attempts to specifically target or tailor smoking cessation services to meet the needs of these communities[27,28]. This is of concern as qualitative work (using in-depth interviews and focus groups), has shown that culture and religion are strong influences on smoking attitudes and behaviours[3]. There was found to be widespread awareness of the dangers of smoking and a high degree of motivation to quit, with many quit attempts; these however tended to rely on "willpower" alone and were (unsurprisingly) typically unsuccessful. Knowledge of, beliefs about and limited access to known effective smoking cessation interventions were shown to be important cultural and structural barriers to improving quit rates. The authors proposed that services should be flexible, multi-lingual, supportive and involve local people in their delivery.

Although many smokers also emphasised the importance of religious centre based services, a telephone survey of a random sample of 50 British mosques found that whilst most would like to support smokers to quit, few Imams (religious leaders) believe that their centres currently have the capacity to support this work making a mosque-based intervention unfeasible at present. Similarly, our enquiries have found that the prospects of a Fatwa (religious edict) – something which qualitative work also suggested might be useful to help smokers quit – is also very unlikely in the near future in Britain (Mogra I, Chair of Mosque and Community Affairs Committee, Muslim Council of Britain, personal communication, 2005) and even in countries where such Fatwas have been issued the impact of these has, at best, been extremely limited[29].

### Rationale for this trial

Our enquiries, the qualitative work already undertaken, and the experiences of services that have begun to develop new styles of smoking cessation service delivery suggest that "grass-roots" trained community smoking cessation worker delivered interventions are likely to prove feasible, accessible and effective.

This study seeks to build on these insights and develop and then pilot a model of community stop smoking services that will be "owned by" and prove acceptable to Bangladeshi and Pakistani male smokers, and their wider communities. Our enquiries indicate that there are several models of care centred on bi- or multi-lingual trained community smoking cessation workers currently in existence. These range, for example, from facilitators focusing on improving access to existing mainstream smoking cessation services to developing home-based specialist parallel services. It is, however, important to prioritise the evaluation of interventions that are potentially generalisable across the NHS hence the focus here is on interventions that have the potential to integrate with existing smoking cessation services.

### Aims and study questions

#### Aim

To develop and investigate the potential of a culturally appropriate community smoking cessation service in improving access to and the effectiveness of smoking cessation services for male adult Pakistani and Bangladeshi smokers.

#### Study questions

Primary study questions:

1. What are the rates at which the population of Pakistani and Bangladeshi male smokers will set a quit date with the stop smoking services in the intervention and control areas?

2. What proportions of those setting a quit date in the intervention and control areas achieve biochemically verified prolonged abstinence from smoking at a) 4 weeks, b) 3 months and c) 6 months after the agreed quit date?

3. What is the likely degree of contamination of the intervention and control areas and the design effects that need to be taken into consideration when conducting sample size calculations for a definitive cluster randomised controlled trial?

4. What are the key components of the intervention as it develops and how do these components relate to the outcome measured by the rates of setting quit dates and abstinence among those setting quit dates?

Secondary study questions:

5. What proportion of Pakistani and Bangladeshi male smokers that book an appointment with the stop smoking service attend the initial appointment and set a quit date in the intervention and control areas?

6. What smoking cessation treatments do Pakistani and Bangladeshi men use in the intervention areas, facilitated by outreach workers, and in the control areas, without such workers?

7. What are the experiences of the services of the relevant groups in each of the trial arms and how satisfied are they?

8. What proportions of those enrolled adhere to the interventions chosen?

9. What impact, if any, are these interventions likely to have on smokeless (oral) tobacco use in these smokers?

10. What are the costs, from a health service perspective, of delivering these interventions?

## Methods

### Study/design

This is a pilot cluster randomised controlled trial being undertaken in Birmingham East and North Primary Care Trust (BEN PCT) and the Heart of Birmingham Teaching Primary Care Trust (HoB tPCT). The trial compares the change in the key outcome variables between intervention and control areas. The change is the difference between the intervention period rates of activity in the intervention area and the same period one year prior to the intervention. An analogous figure is calculated for the control areas and the difference in these changes are then compared.

### Interventions

The interventions being tested in this two arm pilot trial are: (i) standard stop smoking advisor based in smoking cessation clinic ('clinic only' control) *vs*. (ii) community based stop smoking advisor working in an outreach capacity, to support existing clinics enhance the tailoring of smoking cessation treatments to the specific needs of Pakistani and Bangladeshi male smokers and/or to provide smoking cessation treatment themselves in a location of their choosing ('clinic + outreach').

The aim of both models of care is to attract clients, to convince them of the value of behavioural support and pharmacotherapy, to provide these interventions themselves or point people towards clinics providing such treatment, and to persuade clients to persist with treatment until the risk of relapse is substantially decreased.

#### 'Clinic only' (control)

In the areas allocated to the 'clinic only' arm, the NHS stop smoking services will attract clients in the normal way. The model is well developed in BEN PCT and HoB tPCT. The model consists of health care service providers, including general practitioners (GPs), nurses, pharmacists and stop smoking advisors (SSAs) who are trained in smoking cessation, and work to standards set and monitored by the local stop smoking services.

#### 'Clinic + outreach' (intervention)

In areas allocated to the 'clinic + outreach' arm, the NHS stop smoking services will attract clients with additional support from four community based stop smoking advisors, also known as 'outreach workers'. These outreach workers come from the ethnic groups of interest (i.e. two of Bangladeshi origin and two of Pakistani origin) and live in the communities that they serve. Between them, they speak several relevant languages (i.e. Sylheti, Bengali, Punjabi, Mirpuri, Urdu and English).

A specialised training programme for the outreach workers will be organised prior to the start of the intervention, to combine training in smoking cessation with a special understanding of the norms and beliefs about tobacco in the Pakistani and Bangladeshi communities. This will consist of:

◦ Standard two-day SSA training delivered by HoB tPCT

◦ Three-day health promotion training course delivered by HoB tPCT on exploring ways to promote well-being and health, and techniques involved in the planning, preparation and delivery of approaches.

◦ Sessions given by primary care staff at the University of Birmingham on research skills, communication skills, the function of the NHS, the cultural context of the work, and discussion of the outreach role.

We envisage that the outreach workers will take the following approaches to improve the reach of and access to the smoking cessation services:

◦ Undertake community engagement work to help increase awareness of smoking cessation services

◦ Assist existing service providers with the delivery of stop smoking support and promotions

◦ Provide stop smoking support and medication for people in non-clinic venues, e.g. workplaces, public buildings.

Additionally, we envisage that the outreach workers will draw on their in-depth understanding of beliefs about and attitudes towards smoking, in order to:

◦ Raise awareness of the dangers associated with smoking and counsel smokers wishing to quit

◦ Identify suitable quit dates (which may be based on key religious events such as a Friday congregational prayer, journey on Umrah or Hajj (pilgrimage to Mecca), or to coincide with the month of Ramadan, for example)

◦ Highlight religious objections to smoking, where appropriate

◦ Make available existing and custom-made literature, telephone (Asian QuitLine) and other support materials.

We hypothesise that the 'clinic + outreach' model will attract more patients to the service because it is seen as more culturally appropriate. In addition, the service should influence the proportion of people successfully attempting to stop smoking by encouraging patients to persist with treatment.

### Randomisation

We will use census super output areas (SOAs) as the unit of allocation[30]. In order to define these communities, initially all those SOAs within the study area with a combined Bangladeshi and Pakistani population of more than 10% will be identified and then mapped at ward level, enabling the street boundaries of each SOA to be seen. Contiguous SOAs will then be aggregated into natural communities (i.e. areas where people live, work, shop etc) using the local knowledge of the LSSS managers and advisors. SOAs in which more than 30% of the population are Pakistani and Bangladeshi will be considered to be high-density areas, while SOAs with 10–29% of the population being from these groups will be defined as low-density areas.

**Figure 1:**
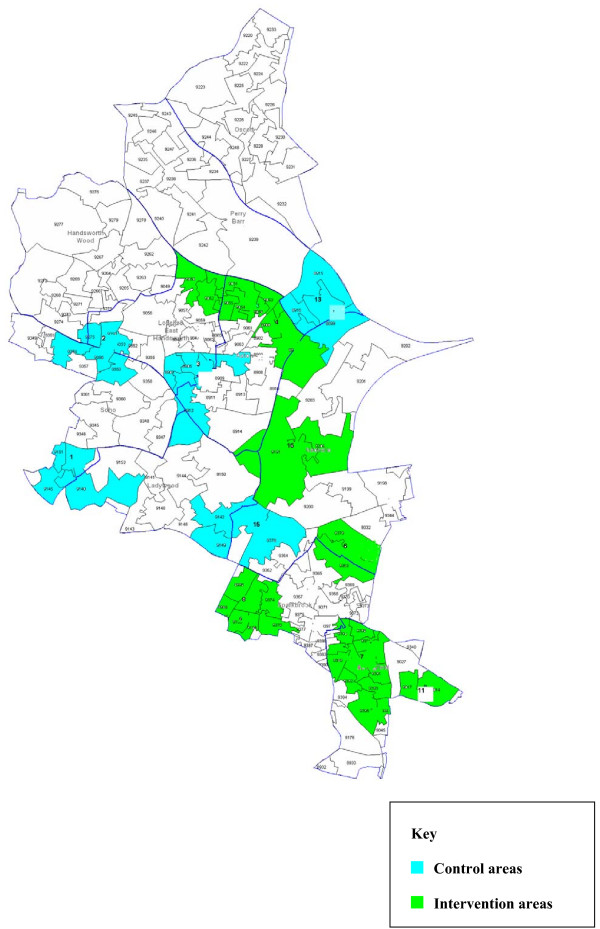
**Map of randomisation areas in HoB tPCT**.

**Figure 2:**
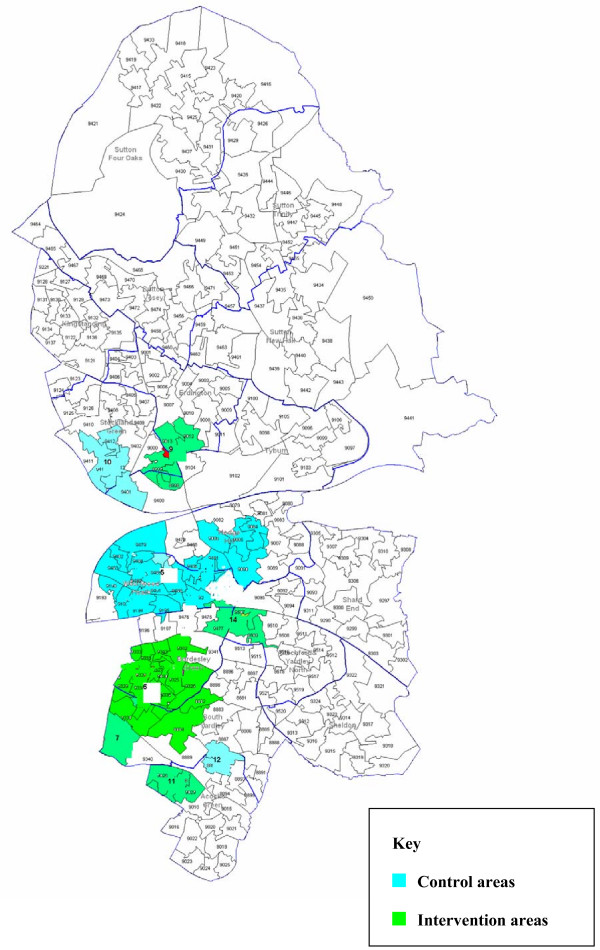
**Map of randomisation areas in BEN PCT**.

A total of 16 aggregated areas, sufficiently geographically dispersed, will be created to be randomised; there will thus be eight SOAs in each arm. For the randomisation, we will stratify, firstly by the proportion of Pakistani and Bangladeshi residents and secondly by absolute population size, to create eight areas that include high target population density and eight that are low-density areas. The high density areas will be further subdivided into those areas with target populations of Bangladeshi and Pakistani men above and below 10,000. The low density areas will be further subdivided into those areas with target populations above and below 300. Table 1 shows how the areas will be allocated. Randomisation will be undertaken by the study statistician who has no knowledge of the geographical areas using permuted blocks. Within each of the four strata, two areas will be randomly allocated to 'control' and two to 'intervention' arms. Maps of the final areas and their allocation to the two study arms are shown in Figures 1 and 2.

**Table 1:** Randomisation allocation

Number of areas	Bangladeshi and Pakistani population density	Bangladeshi and Pakistani population size	Study arm
2	High*	<10,000	Clinic only

2	High	<10,000	Clinic + outreach

2	High	>10,000	Clinic only

2	High	>10,000	Clinic + outreach

2	Low**	<300	Clinic only

2	Low	<300	Clinic + outreach

2	Low	>300	Clinic only

2	Low	>300	Clinic + outreach

*Pakistani and Bangladeshi populations = >30%

** Pakistani and Bangladeshi populations = 10–29%

In studies in which the number of units randomised is relatively small, stratified randomisation of communities should ensure equal distribution of key potential confounders. However, potential confounders are also important contextual factors affecting the success or otherwise of the interventions and thus possible effect-modifiers. We will therefore gather data on relevant aspects of the communities (in particular socio-economic position, since this is a strong predictor of smoking status) participating in the trial. Such data will be critical in enabling generalisation of the lessons learnt from this pilot trial.

### Protecting against sources of bias and reducing contamination

The main source of bias in this trial is likely to arise from contamination (i.e. people helped to stop smoking with the aid of the LSSS who live in 'clinic only' areas). The potential for contamination will be reduced by careful planning. First, natural communities will be chosen – places where local intelligence tells us people tend to live *and *work. Second, buffer zones will be created around the trial areas that are neither 'clinic only' or 'clinic + outreach' in order to reduce the possibility of people living in control areas crossing into intervention areas and coming across intervention workers (see Figures 1 and 2). This will mean excluding large areas that have both high and low densities of Pakistani and Bangladeshis living in them that would otherwise have participated in the trial. We will be able to gauge the degree of contamination by using a before-after design. Working with anonymised versions of the LSSS databases, we will analyse data on the use of services by Pakistani and Bangladeshi residents of intervention and control areas and other areas of Birmingham mostly distant from our intervention and control areas. We will thus be able to contrast the change in service use, not simply differences in service use. Contamination would be marked by increases in service use in areas assigned to either the control or special intervention that would not be seen in outside areas that served as the external control. Informal conversations with patients, service providers, and outreach workers will also be carried out to provide additional insight into the extent of any contamination.

The two quantitative primary outcomes are uptake and abstinence rates. Nearly all people contributing data to these rates will be unaware of the trial. Thus the potential for information bias is minimal.

The first three primary study questions will be answered using LSSS data, most of which are collected routinely (LSSS Dataset). The fourth primary question will be answered by a qualitative study, consisting of longitudinal focus group discussions with the outreach workers, observation of their management meetings, analysis of their diaries, and some shadowing of their activities.

**Figure 3:**
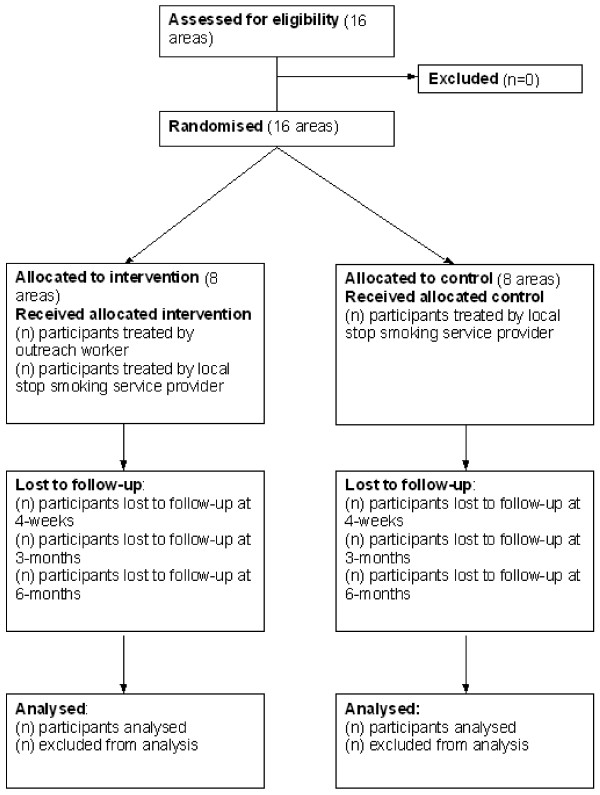
**Flow diagram of clusters and participants in the trial**.

The secondary questions will be addressed either through LSSS data on treatments used, or through an Extended Dataset collected from a self-selected sub-sample of participants in the trial (Extended Dataset). Data sources and outcome measures for each of these research questions are summarised in Table 2. The flow of participants through the trial is shown in Figure 3.

**Table 2:** Research questions and associated trial outcome measures

Research question	Outcome measure	Data source
**LSSS Dataset**		

1. What is the rate at which the population of Pakistani and Bangladeshi male smokers will set a quit day with the stop smoking services in the intervention and control areas.	Recruitment rate is defined as the number of relevant population setting a quit day with the services as a fraction of all those estimated to have attempted to stop.	LSSS routine database

2. What proportions of those setting a quit day in the intervention and control areas achieve prolonged abstinence from smoking at a) 4 weeks, b) 3 months and c) 6 months after the agreed quit day?	a) Number of quits (measured by self assessment questionnaire & CO monitoring) Total number of smokers* accessing LSSS b) & c) number of quits (measured by self assessment questionnaire & CO monitoring)	a) LSSS routine database b) & c) LSSS data collected specifically for the trial

3. What is the likely degree of contamination of the intervention and control areas and the design effect that need to be considered when conducting sample size calculations for a definitive cluster randomised controlled trial?	The design effect from cluster randomisation will be calculated from the multilevel analysis. Contamination cannot be easily measured; however, we have external controls which will help indicate whether it is occurring.	

4. What are the key components of the intervention as it develops and how do these components relate to the outcome measured by the rates of setting quit dates and abstinence among those setting quit dates?	Qualitative data	Focus groups with outreach workers Observation of management meetings Shadowing Analysis of outreach worker diaries End of project interviews with outreach workers

5. What proportion of Pakistani and Bangladeshi male smokers that book an appointment with the stop smoking service attend the initial appointment and set a quit day in the intervention and control areas?	To be estimated from a sample of service providers records	SSA records LSSS Routine database

**Extended Dataset**		

6. What smoking cessation treatments do Pakistani and Bangladeshi men use in the intervention areas, facilitated by outreach workers, and in the control areas, without such workers?	Numbers of clients choosing each available treatment option (e.g. NRT, Zyban etc)	LSSS Routine database Patient satisfaction questionnaire administered by LSSS 'Call to Quit' team

7. What are the experiences of the services of the relevant groups in each of the trial arms and how satisfied are they?	Patient satisfaction	Patient satisfaction questionnaire administered by LSSS 'Call 2 Quit' team

8. What proportions of those enrolled adhere to the interventions chosen?	Numbers using oral tobacco during quit attempt (self report) Number of people adhering to each medication used in each arm of the trial (self report) Attendance at clinic in each arm of the trial	Adherence rating week by week then averaged, collected by SSA on extended data monitoring form

9. What impact, if any, are these interventions likely to have on smokeless (oral) tobacco use in these smokers?	Numbers using oral tobacco during quit attempt (self report)	Frequency rating week by week, collected by SSA on extended data monitoring form Frequency of use of oral tobacco during this quit attempt rating on Patient satisfaction questionnaire administered by LSSS 'Call to Quit' team

10. What are the costs, from a health service perspective, of delivering these interventions?	Estimated benefits of the intervention in terms of QALYs or LYGs.	Additional costs to NHS of (i) Employment, training, management of outreach workers- (ii) Extra resources used by LSSS – e.g. medication, behavioural support (Calculated from estimates of extra time used)

### Local Stop Smoking Service (LSSS) Dataset

#### Inclusion criteria

Any male (≥18 years) Pakistani or Bangladeshi using the NHS stop smoking services in the study areas will be eligible for inclusion in this study.

The LSSS routinely records abstinence rates at four weeks post quit date. Anyone from the target population who maintains abstinence at four weeks will be eligible for follow-up at three months. Service users will be contacted by the Birmingham 'Call 2 Quit' line – a special project to enhance the stop smoking services in Birmingham – and invited to attend a clinic appointment for verification of abstinence using expired carbon monoxide. Any service users who are still abstinent at three months will be followed up in the same way at six months.

#### Exclusion criteria

Any users of the NHS stop smoking services who are not from the relevant ethnic groups will be excluded. Anyone from the target population who does not maintain abstinence at four weeks will not be eligible for follow-up at three or six months.

#### Consent

Consent from patients will not be required as the data will be received from the Primary Care Trusts (PCTs) in an anonymised form.

#### Losses to follow-up

Loss to follow-up is not an issue in relation to the primary outcomes of use of services or abstinence. People who attend a stop smoking service set a quit date at one point in time or they do not, and only those who do are monitored by the LSSS. Furthermore, the other important outcomes (i.e. cessation at four weeks, and three and six months) are routinely monitored by the service too. As is standard protocol of smoking cessation research, people who are lost to follow-up are deemed to be smoking again[31,32].

### Extended Dataset

The aim of the Extended Dataset is to provide more detailed data on attendance and adherence than can be obtained from the NHS records and to provide information on service use satisfaction that cannot be obtained from NHS records. To accomplish this, Pakistani and Bangladeshi men using particular NHS services will be approached and asked for consent to provide this information to the research team.

#### Recruitment

A sub-sample of individuals who go through the NHS LSSS in these areas will be invited into a study of their perceptions of the service. We will recruit providers of NHS stop smoking services in both our study areas to collect this additional data. These will be drawn from those stop smoking advisors, nurses and pharmacists who provide smoking cessation services in or near the study areas that have, on past data, delivered stop smoking services for Pakistani and Bangladeshi men residing in the intervention or control areas. We will supply each SSA taking part in the extended data collection with a pack containing a brief procedure guide, information sheets, consent forms, data collection forms and a method to contact the study team. The baseline patient questionnaire incorporates items from a culturally valid tobacco use questionnaire[33]. Service users will be asked to participate by their service provider (outreach worker, nurse, specialist SSA or pharmacist) during routine consultation. These data will be supplied to the study team confidentially, but not anonymously.

#### Inclusion criteria

All consenting, self-assigned (using Census 2001 categories)[5] Pakistani and Bangladeshi male adult (≥18 years) regular smokers (defined as self-declared usage of on average ≥1 cigarette/day) using the NHS stop smoking services will be eligible to participate.

#### Exclusion criteria

Any users of the NHS stop smoking services who are not from the relevant ethnic groups will be excluded.

#### Consent

Written consent will be obtained from all users prior to their inclusion in this extended data collection phase.

#### Losses to follow-up

Those individuals recruited in the Extended Dataset will be asked about their satisfaction with the stop smoking service they received. It is the experience of LSSS that individuals who return to smoking do not return to clinic, while those who have succeeded do so and are typically very pleased with the care they received. Thus, loss to follow-up is a potentially major bias in assessing these outcomes. However, these individuals will have consented to follow up and thus we seek to minimise loss of information from those who do not stop smoking by telephone contact from the Birmingham Call 2 Quit line.

### Embedded qualitative work

A key aim of phase II of the MRC Complex Intervention Framework is to develop and test out interventions. We will conduct longitudinal qualitative work to help define the fundamental components of the intervention and to develop a step-by-step account of how the intervention evolves over time. The project researcher will attend monthly working meetings between the outreach workers and the management team to record discussions about activities undertaken, the response of the population to them, and changes made to work plans as they emerge. Furthermore, the outreach workers will be encouraged to keep detailed weekly records of what they do, which will form part of their regular progress reports to their supervisors at the stop smoking services. The project researcher will also shadow the outreach workers, making field notes while they engage in outreach activity on the streets and during promotional events and clinic sessions. Focus group discussions with the outreach workers will be carried out at quarterly intervals during data collection in order to:

◦ Define more clearly the role of the outreach worker in assisting Bangladeshi and Pakistani smokers to stop smoking

◦ Explore the approaches taken when recruiting service users and supporting smoking cessation and why they chose those particular approaches

◦ Understand how the service provided changes over time, as their role, and what does and does not work, becomes clearer.

One-to-one interviews with individual outreach workers will be carried out at the end of the study to capture any differences in ways of working.

We also aim to capture what happens in the pre-existing clinic based intervention (pharmacies), in order to be able to record how different the 'clinic + (outreach)' intervention is in terms of approaches to cessation support. Whilst it is often expected that there is a standard approach, in practice there is often surprisingly large variation. To this end we will carry out a small number of brief one-to-one interviews with a purposive sample of pharmacists in the study area to develop a clearer picture of any added value of the outreach worker intervention.

### Risks to the safety of service users in the trial

There are no anticipated risks to clients from participation in this study over and above those associated with using a smoking cessation service. Most participants will not even know that they are participating, as their routinely collected NHS data will be passed in anonymised form to the researchers. For patients participating in the Extended Dataset, there is the risk of inadvertent disclosure that they smoke to relatives that do not know this; however the study team are aware of this and will minimise this risk.

### Outcome measures and analysis

#### Proportion of people attending but not setting quit dates

Stop smoking services return only the number of people setting quit dates with the service, so these data are not routinely collected. To obtain these, we need to identify service providers in intervention and control areas that keep meticulous booking records. If we find such providers during the collection of the Extended Dataset, we will try to estimate the proportion of people who attend without setting a quit date. However, it is unlikely that booking records give the ethnic group of the person booking, so this may prove impossible. Should appropriate data be available, the number of quit days set in relation to the number of bookings will be examined using a Poisson multilevel model for intervention and control areas.

#### Denominator for the proportion of smokers setting quit dates

Our overall aim is to develop and refine the intervention, to pilot it, and have the data needed to inform sample size calculations for a definitive trial evaluating these interventions. In order to perform sample size calculations for a future trial, we aim to assess the reach of the interventions in attracting smokers wanting to quit. For the purposes of this study, reach has been defined as the proportion of each relevant ethnic group setting a quit date using the stop smoking service, with the denominator for this being all those of relevant ethnic group attempting to quit during the same period. We are estimating this latter number as it cannot be directly measured. We have searched a variety of sources to give information on the proportion of each ethnic group that attempted to quit in the past year and these data are not available. No local survey can truly randomly survey Pakistani and Bangladeshi populations because there is no publicly available local sampling frame that records ethnicity. Consequently, we will use the Smoking Toolkit Study to provide relevant data on the proportion of people that attempted to quit in the past year for the general population and assume that this figure is approximately the same in Pakistani or Bangladeshi men[34].

Stop smoking services routinely record information about each of their patients, including their age, gender, ethnic group, type of service used, medication used and outcome. This will be used to give both a before and after measure of the use of the service by the relevant groups (i.e. males of Pakistani and Bangladeshi ethnic group). Thus, we will have, for each area, the rate of use of the service for 12 months prior to the intervention that will serve as a baseline variable in the analysis (see Table 3). The after measure will be the same data collected for the 12 months during which the outreach workers are active. These data will be supplied anonymously from BEN and HoBt PCTs and this does not rely on data collection developed for the study. As noted above, the before-after design will allow control for intrinsic variability between areas. As such there will be no attempt to assess baseline balance between the intervention and control areas.

**Table 3:** Use of LSSS by randomisation area residents for the baseline period (1st November 2006 – 31^st ^October 2007)

Area number	Number of Pharmacies	Numbers using any LSSS service 06/07	Number of smokers using LSSS within area they live	Number of smokers using LSSS in other areas of randomisation	Number of smokers* using LSSS users out of study zone	Number of smokers using unidentified LSSS
1	0	7	0	0	7	0

2	1	10	1	0	9	0

3	0	29	0	8	20	1

4	3	79	41	4	32	2

5	5	155	57	2	77	19

6	4	136	43	5	84	4

7	6	98	55	0	42	1

8	2	6	0	1	5	0

9	1	2	0	0	2	0

10	0	4	0	0	3	1

11	0	8	0	5	3	0

12	0	3	0	0	3	0

13	0	11	0	8	2	1

14	1	8	0	3	5	0

15	0	2	0	0	2	0

16	0	5	0	0	5	0

*Bangladeshi or Pakistani Males aged 18 years or over

Although we will estimate the proportion of people setting a quit date, we cannot truly know the denominator, as in many cases people set multiple quit dates, hence we will consider this a rate and the 'denominator' an offset in the analysis.

Quantitative analysis will use a multilevel approach. In this model the outcome variable will be the number of individuals setting a quit date in each of the 16 randomised areas. The quasi-denominator is the estimated number wishing to quit in each area, and the log of this number will be used as an offset in a Poisson regression model. Independent variables in the model will be the group (control or intervention) and the log of the numbers setting a quit date in the previous 12 months before the trial. The randomised areas will be included in the model as a random effect, thereby allowing for the clustering inherent in the design[35]. This will be used in estimating the intra-class correlation coefficient, which in turn will determine the design effect. This is the multiplier which has to be applied to standard sample size formulae to allow for the effect of randomising at the cluster level instead of the individual level[36]. The stratification used in producing the randomisation will not be utilised in the analysis.

#### Definition and modelling of abstinence rates

The quit rate is defined as the proportion of people achieving abstinence, with a denominator of all those who attended the service and set a quit date. Quitting is defined as prolonged abstinence, which is defined as allowing lapses during the first two weeks of the quit process measured according to the Russell standard[37]. Self-reporting is substantiated by biochemical validation using expired carbon monoxide (less than 10 parts per million)[38].

The modelling will follow the same approach as described above for calculation of usage rates, except that we will have a true denominator. We will therefore use multilevel logistic regression models rather than Poisson models.

#### Smoking cessation treatments chosen

The proportion of LSSS clients choosing each available treatment option will be calculated from the information provided in the routine stop smoking service.

#### Patient experiences

We have developed a patient satisfaction questionnaire, which will be used to collect information on the experiences of service users. This will be examined for the intervention group using appropriate multilevel models.

#### Estimating adherence

The standard forms for recording the clinical contact with patients record whether the person is using nicotine replacement therapy (NRT) or not, but not degree of adherence, which is quite complicated and impractical for NHS to measure accurately. We will therefore use a measure that defines good or less than good adherence, which is defined for each type of treatment. Stop smoking advisors will be asked to record adherence weekly on a modified routine monitoring form.

This will be modelled with random effects logistic regression, with the numerator being the number of people who adhere well to treatment in each week and the denominator being all those receiving treatment. Repeated measures on the same individuals are correlated within individuals and will be accounted for in the analyses.

#### Estimation of costs

We will approach this using the models developed for other purposes (e.g. the National Institute for Health and Clinical Excellence (NICE) reports on NRT[39]). Thus, if long-term abstinence can be estimated, we should be able to use this to derive an estimate of the benefits of the intervention in terms of Quality Adjusted Life Years (QALYs) or Life Years Gained (LYGs). We will therefore estimate the additional costs of:

◦ The employment, training, and management of the outreach workers

◦ The extra resources used, including extra medication, extra behavioural support given by other providers as a result of additional activity generated by the outreach workers. We will calculate this from the economic costs to the NHS estimated from time used and/or observations of extra time taken.

#### Qualitative work

A modified Framework approach will be used systematically to make sense of the different types of qualitative data that will be generated[40]. Issues that emerge from these data will be indexed and collated to form broad categories. The categories will be examined and emergent themes identified in regular discussions of the multi-disciplinary qualitative sub-group, with emerging ideas being incorporated into future rounds of the qualitative data collection.

### Current study status

The intervention period began on 1^st ^November 2007 and ran for 12 months, with an end date of 31^st ^October 2008. Final data collection and analysis of data in relation to the primary outcomes is currently underway and we anticipate will be completed by July 2009.

#### Protocol amendments

From the time of writing this protocol, the following changes have been made.

We aimed to measure the rate at which Pakistani and Bangladeshi male smokers set a quit date with the stop smoking services in the intervention and control areas. We defined reach as the proportion of each relevant ethnic group setting a quit date using the stop smoking service, with the denominator for this being all those of relevant ethnic group attempting to quit during the same period. As this latter number could only be estimated, we investigated whether The Health Survey for England ethnic boosted sample data had the relevant information (i.e. the proportion that tried to stop in the previous year). These data did not, however, contain this information and we have therefore assumed that the proportion of Pakistani and Bangladeshi males that attempt to quit is the same as the proportion of all other ethnic groups. This assumes that what is true of the majority population is true of Pakistani and Bangladeshi populations.

Prior to the start of the study, the randomisation of the intervention and control areas were switched, as the initial allocation meant more Pakistani and Bangladeshi target populations resided in areas allocated to the control than intervention. The managers of HoB and BEN PCTs preferred that the outreach workers were given the opportunity to reach more people, thus, the randomisation was switched in consultation with the trial statistician and Independent Project Steering Committee.

Initially, we aimed to follow-up LSSS service users who achieved 4-weeks prolonged abstinence from smoking at three months from the agreed quit date. The Birmingham 'Call 2 Quit' line was unable to do this at three months. However six-month follow-up proceeded as planned.

### Ethical approval

This study has been approved by South Staffordshire Local Research Ethics Committee.

## Abbreviations

BEN: Birmingham East and North Primary Care Trust; HoBt: Heart of Birmingham Teaching Primary Care Trust; LSSS: Local Stop Smoking Services; LYG: Life Years Gained; NICE: National Institute for Health and Clinical Excellence; NRT: Nicotine Replacement Therapy; PCTs: Primary Care Trusts; PHIT: Public Health Information Team; QALY: Quality Adjusted Life Years; RS3: Re-designing Stop Smoking Services; SOA: Super Output Area; SSA: Stop Smoking Advisor; SSS: Stop smoking Services.

## Competing interests

Paul Aveyard has done consultancy work for Pfizer, McNeil, and Xenva (now Celtic) Biotechnology with regard to smoking cessation. All other authors declare that they have no competing interests.

## Authors' contributions

AS and RSB conceived this study and together led the bid to secure funding for this work and manage the project. AS, PU, and PA developed the trial protocol. PU, RAB, PA, and AS managed the study. RSB, PG, MW, AA, RJP, RB, PMB, QZ and MF all contributed to designing the study and overseeing its implementation. PU and RAB were the researchers employed on this project and led the drafting of this paper. All authors commented on draft versions of this manuscript and approved the final version prior to submission.
